# Physical Deconditioning in Lung Cancer Patients Who Underwent Lung Resection Surgery in Spain: A Prospective Observational Study

**DOI:** 10.3390/cancers16162790

**Published:** 2024-08-08

**Authors:** Alejandro Heredia-Ciuró, Florencio Quero-Valenzuela, Javier Martín-Núñez, Andrés Calvache-Mateo, Geraldine Valenza-Peña, Laura López-López, Marie Carmen Valenza

**Affiliations:** 1 Department of Physiotherapy, Faculty of Health Sciences, University of Granada, 18071 Granada, Spain; ahc@ugr.es (A.H.-C.); javimn@ugr.es (J.M.-N.); andrescalvache@ugr.es (A.C.-M.); geraldinevalenza@ugr.es (G.V.-P.); cvalenza@ugr.es (M.C.V.); 2 Thoracic Surgery Unit, Hospital Virgen de las Nieves de Granda, 18014 Granada, Spain

**Keywords:** lung cancer, pulmonary surgical procedures, pain, physical fitness, dyspnea

## Abstract

**Simple Summary:**

Lung-resected patients experience physical deterioration that limits their quality of life, but there are important gaps in the knowledge of the evolution of this deterioration. The aim of this study was to assess physical deterioration in lung cancer survivors in the short and medium term, using self-administered functional measures that would allow us to obtain information about patients’ perceptions. These results can facilitate the future management of lung cancer patients after resection, reducing the sequelae they suffer and improving their quality of life.

**Abstract:**

Background. Lung resection represents the main curative treatment modality for lung cancer. These patients present with physical deterioration that has been studied previously using objective variables; however, no previous studies have evaluated the self-perceived physical fitness of these patients. For these reasons, to increase the current knowledge on lung cancer patients’ impairment, the aim of this study was to characterize the self-perceived physical deconditioning of lung cancer patients undergoing lung resection in the short and medium term after surgery. Methods. A longitudinal, observational, prospective cohort study was performed in the Thoracic Surgery Service of the Hospital Virgen de las Nieves (Granada). Symptoms (pain, fatigue, cough and dyspnea) and physical fitness (upper and lower limbs) were assessed before surgery, at discharge and at one month after discharge. Results. Among the total of 88 patients that we included in our study, significant differences were found at discharge in symptoms (*p* < 0.05) and physical fitness (*p* < 0.05). One month after surgery, higher levels of pain (*p* = 0,002) and dyspnea (*p* = 0.007) were observed, as well as poorer results in the upper (*p* = 0.023) and lower limbs’ physical fitness, with regard to the initial values. Conclusions. Patients undergoing lung resection present an increase in symptoms and physical fitness deterioration at discharge, which is maintained one month after surgery.

## 1. Introduction

Lung cancer is one of the most prevalent cancers worldwide [1], being the deadliest cancer entity in males and the second in females [2,3]. Non-small cell lung cancer (NSCLC) accounts for 80% of lung cancer cases [4], representing the most prevalent lung cancer entity in recent years.

Surgical tumor resection remains a prerequisite for a cure and extended survival; for this reason, lung resection serves as the primary treatment for these patients [5]. Despite the improvement in surgical techniques in recent years, lung resection is still associated with a high incidence of post-operative complications that extend hospital stays and slow physical recovery after intervention [6,7].

Lung resection implies tissue damage that disturbs pulmonary and cardiovascular systems [8,9], provoking pain [10], respiratory muscle damage [11] and loss of muscle strength [12]. These factors have been associated with a limitation in exercise capacity and a physical decline in these patients [10].

The published literature [13,14] has also related cancer entities and cancer treatment with a reduction in physical functioning. This physical impairment, added to cancer-related symptoms [15,16,17], has been shown to disrupt the daily functioning and quality of life of lung cancer patients, impacting the incidence of post-operative complications and increasing their morbidity and mortality [18,19,20].

Previous studies [21,22] have evaluated this physical impairment of lung cancer surgical patients. However, most of the studies have used direct measures such as VO_2_ peak [23], and no previous studies have applied self-administered functional measures, which provide information about patient perception. In this sense, submaximal exercise capacity tests reflect the physical functioning and self-perceived effort of patients [24,25,26] through the dyspnea and fatigue expressed during the test.

Sustaining optimal physical function and controlling symptoms after surgical resection could improve the functionality and quality of life of lung cancer patients [27,28]. Therefore, to enhance the current understanding of the impairments experienced by lung cancer patients, this study aimed to characterize the physical deconditioning of lung cancer patients undergoing lung resection both immediately after surgery and in the following month.

## 2. Materials and Methods

A prospective observational study was carried out between October 2019 and July 2022. Lung cancer patients who were undergoing lung surgical resection were recruited from the Thoracic Surgery Service of the Hospital Universitario Virgen de las Nieves de Granada (HVN). This study adhered to the Declaration of Helsinki and followed the STROBE guidelines throughout the research process [29]. The study protocol was reviewed and approved by the Biomedical Research Ethics Committee of Granada (Granada, Spain).

Patients were included if they met the following inclusion criteria: (1) lung cancer survivors, (2) aged 18–80 years, (3) candidates for lung resection, (4) informed about the study purpose and (5) signed the informed consent. The exclusion criteria were diseases or conditions that prevented the proper execution of the tests or assessments conducted in the study, such as cognitive impairment, mental instability, or neurological pathologies.

Patients were evaluated pre- and post-surgery and at 1-month follow-up by pre-trained investigators. All patients adhered to a standardized recovery protocol: post-lung surgery, they spent 24 h in the post-anesthesia care unit and received uniform analgesic treatment, primarily non-steroidal anti-inflammatories, throughout their hospitalization. Upon confirmation of inclusion criteria, a structured interview and initial assessment were performed. Relevant medical history data, such as anthropometric measurements, comorbidities (assessed using the Charlson comorbidities index) [30] and the duration of the operation, were also collected.

The main outcomes included cancer-related symptoms and upper and lower limb exercise capacity.

### 2.1. Cancer-Related Symptoms

Cancer-related symptoms included dyspnea, pain, cough and fatigue.

Dyspnea. The Borg-modified scale was used to assess dyspnea, which has been validated in both cancer and respiratory patients [28]. Patients indicated their level of respiratory distress on a scale ranging from 0 to 10, where 0 denoted no distress, and 10 indicated severe difficulty in breathing.

Pain. Pain levels were evaluated using the Brief Pain Inventory (BPI), a validated tool designed to gauge both pain intensity and its impact on daily life in cancer patients [31,32]. Patients rated the severity of their pain at its peak, minimum, and current levels over the past week. Additionally, they assessed pain interference across seven contexts, including work, activity, mood, enjoyment, sleep, walking and relationships. The BPI has demonstrated strong reliability and validity through extensive psychometric testing [33].

Cough. Cough was assessed with the Leicester Cough Questionnaire (LCQ) [34], a questionnaire translated and validated into Spanish by Muñoz G. et al. [35] that measures the impact of cough on patients’ lives. The LCQ contains nineteen items with scores on a Likert scale ranging from 1 to 7. This scale presents three domains where cough impact over the prior 2 weeks is assessed physically, psychologically and socially. The score ranges from 3 to 21, where a lower LCQ score indicates a worse cough.

Fatigue. Fatigue was evaluated with the fatigue severity scale (FSS). The FSS [36] was developed to measure the impact of disabling fatigue on daily functioning and the severity of the presented fatigue. The instrument consists of nine items on a Likert scale that range from 1 (strongly disagree) to 7 (strongly agree). The total score ranges between 9 and 63. A higher score indicates more self-perceived fatigue.

### 2.2. Upper Limb Exercise Capacity

The upper limb exercise capacity was evaluated by handgrip strength and unsupported upper limb exercise tests.

Handgrip strength. Handgrip strength is a reliable marker of peripheral muscle strength [37]. To measure it, a handgrip dynamometer (TEC-60; USA) was employed, and participants were instructed to perform three repetitions using their dominant hand, with the peak force recorded in Newtons. The test was conducted with the patient seated, their shoulder adducted with neutral rotation, their elbow flexed to 90°, and their forearm in a neutral position.

Unsupported upper limb exercise test (UULEX). The unsupported upper limb exercise (UULEX) test, developed by Takahashi et al. [38], is a progressive evaluation intended to measure the maximum capacity for unsupported arm exercises. During the test, participants lifted a bar from their lap to their highest attainable height until they could no longer continue. The score is based on the total time recorded in seconds. Furthermore, the participants’ self-reported dyspnea and lower limb fatigue were assessed using a modified version of the Borg scale [39].

### 2.3. Lower Limb Exercise Capacity

The lower limb exercise capacity was evaluated by lower limb strength and Five Times Sit-to-Stand tests.

The lower limb strength was evaluated using a handheld dynamometer (Lafayette Manual Muscle Testing System, model 01163, Lafayette, IN, USA) [40]. The assessment was conducted with the patient seated, with both knees and hips bent at a 90° angle. Resistance was administered to the knee extension, requiring a maximal muscle contraction for a duration of 5 s. Three repetitions were conducted on the dominant leg, and the maximum value recorded in Newtons was selected for analysis.

Five Times Sit-to-Stand (5STS). The Five Times Sit-to-Stand (5STS) test has been utilized in prior studies to assess exercise tolerance among respiratory patients [41]. Participants were instructed to rise to a full standing position and then sit down firmly, repeating this sequence five times consecutively without utilizing their upper limbs, with the duration recorded as the participant’s score. Additionally, participants’ self-reported levels of dyspnea and lower limb fatigue were documented using a modified version of the Borg scale [39].

Statistical analyses were performed using IBM SPSS Statistics 20.0 software for Windows (SPSS Inc. and IBM Company, Chicago, IL, USA). Descriptive statistics (mean ± SD) or percentages (%) were used to describe sample baseline characteristics. The Kolmogorov–Smirnov test was performed to assess continuous data normality prior to statistical analysis. Differences between different outcomes pre-and post-surgery, as well as at 1-month follow-up, were analyzed using the Paired Samples *t*-test. A 95% confidence interval was applied for statistical analysis, and a significance level of 0.05 was set for all tests.

## 3. Results

**Figure 1:**
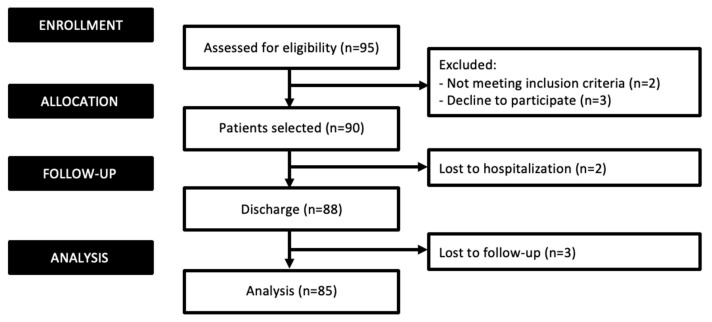
Flow diagram for distribution of participants.

Of the 95 potential patients, 90 were considered eligible and met the inclusion criteria. However, after two losses during hospitalization, 88 patients finally agreed to participate in this study and were evaluated. All participants completed both the pre- and post-surgical evaluations; however, three patients were lost at the one-month follow-up. Figure 1 shows the flow diagram of the participants.

The baseline characteristics of the sample are described in Table 1. The mean age of the participants was 59.33 years, and the percentage of men (60.2%) was higher than that of women (39.8%). The mean body mass index was 26.84 kg/m^2^, and the Charlson index presented a mean of comorbidities of 4.55. The mean surgery duration was around 206.18 min, and the hospital stay was around 6.82 days. The mean value of the exhaled flow volume in the first second was 80.44% of the predicted value. The majority of the sample were ex-smokers (65.7%). The type of tumor that was more prevalent was adenocarcinoma (46.5%), and the majority of the resection was carried out by lobectomy (56.8%) and Video-Assisted Thoracic Surgery.

Analytic values pre- and post-surgery are shown in Table 2.

As seen in Table 2, there are significant differences between pre- and post-surgery in the red blood cells, hemoglobin, leukocytes and pCO_2_, with the pre-surgery values being better (*p* < 0.05).

Table 3 shows the pre- and post-surgery differences in symptoms and physical fitness. As seen, there are significant differences in pain (*p* < 0.001), cough (*p* < 0.001), fatigue (*p* = 0.004) and dyspnea (*p* = 0.013), with the values worsening after surgery.

With respect to the unsupported upper limb exercise test, significant differences were found in the time spent performing the test (*p* < 0.001), although dyspnea and fatigue post-tests showed no significant pre–post differences (*p* > 0.05).

Physical capacity showed significant differences in the strength assessment for the upper (*p* < 0.001) and lower limbs (*p* = 0.001). Significant differences were also found in the time spent performing the 5STS (*p* < 0.001) as well as in post-test dyspnea (*p* = 0.001). However, there were no significant differences in post-test fatigue (*p* > 0.05), although patients showed increased fatigue levels after the intervention.

Table 4 shows the differences in symptoms and physical fitness between pre-surgical and 1-month follow-up. As seen, significant differences were found in dyspnea (*p* = 0.007), pain intensity (*p* = 0.002) and pain interference (*p* = 0.005), exhibiting that participants did not recover their pre-surgical status. However, the fatigue showed a significant improvement one month after the surgery (*p* < 0.001). No significant differences were observed for cough (*p* > 0.05).

Concerning the physical fitness results, there was a significant decrease in the upper limb strength one month after surgery with respect to the pre-surgery status (*p* = 0.001); however, no significant differences were found in the leg dynamometry (*p* > 0.05).

The UULEX showed a significant decrease in the time spent (*p* = 0.023); however, no significant differences were found for dyspnea and fatigue (*p* > 0.05), although patients reported higher dyspnea levels. The 5STS did not show significant differences in the time spent, dyspnea, or fatigue (*p* > 0.05); however, a longer spent time and higher fatigue levels were found when the pre-surgical status was compared to the one-month-after-surgery status.

## 4. Discussion

This study aimed to characterize the physical deconditioning of lung cancer patients undergoing lung resection. Our findings show poor physical recovery with significant symptom extenuation and a significant decline in physical fitness at discharge and in the following month. These findings mark significant progress in the recovery process of lung cancer surgery, as they enable the development of tailored rehabilitation programs for these patients. The participant sample in this study is representative of the broader population undergoing lung resection, reflecting comparable sociodemographic characteristics [42,43].

Cancer-related symptoms of lung cancer surgical patients showed significant exacerbation after surgery, persisting pain and dyspnea after one month. Previous studies carried out in the United States [44,45] have reported similar conclusions to ours, reflecting that some post-surgical impairments could be maintained for 24 months post-resection. Post-surgical pain and dyspnea are two main factors to take into account after cancer treatment because of their importance as predictors of survival in the long term [46].

Concerning physical fitness, a significant decline in strength and exercise capacity was observed, especially in the upper limbs. Previous studies carried out in Germany and India [43,47] also reported a decline in exercise capacity during the first month after lung resection. However, these studies assess the global exercise capacity of lung cancer patients [23] without being specific on upper or lower limb impairments. To the best of our knowledge, there are no previous studies analyzing the impairment of upper limb exercise capacity after lung resection; however, previous studies in similar populations, such as patients undergoing breast cancer or cardiac surgery [48,49], have shown a decline in functionality and exercise capacity after surgery similar to our results.

The strength assessment showed a significant decline in both upper and lower limbs at hospital discharge, maintained for one month after the intervention. Other patients with respiratory pathology who were hospitalized suffered a significant decline in strength, similar to our results [50]. These results should be highlighted due to the value of grip strength as a predictor of functionality [50], mortality and the length of hospital stay [51].

Our study presents some limitations and strengths to be mentioned. A limited sample size was presented due to the difficulty in recruitment to meet the inclusion criteria. However, our sample size is similar to other studies in the lung cancer population [52]. In addition, Propensity Score Matching would have been useful to clarify the homogeneity of the sample. Secondly, a longer follow-up period could have been useful to improve the knowledge about medium- and long-term impairment; however, the characteristics of lung cancer evolution limited the follow-up of these patients.

The strength of our study is the use of self-administered functional measures. Self-administered functional measures are crucial in both clinical research and practice because they provide a direct, efficient means for patients to report their functional status and quality of life. These measures empower patients by allowing them to convey their experiences and challenges without the need for time-consuming and potentially biased clinician-administered assessments [17]. They have been shown to enhance the accuracy and reliability of outcome data, as they minimize the risk of data distortion that can occur when a third party is involved in the reporting process [53]. Moreover, self-administered functional measures are cost-effective and can be easily integrated into routine care, facilitating the regular monitoring of patient progress and treatment impact [54]. By capturing patient-reported data in a streamlined manner, these measures contribute significantly to patient-centered care and help ensure that treatment decisions are informed by the patient’s own perspective [55].

Future studies should include a longer follow-up to obtain more precise data on chronic physical deterioration and its relation to the adjuvant treatments received by these patients. Additionally, more operating data should be collected to try to better stratify patients and understand which variations could mainly affect the results shown. Future lines of research may also propose therapeutic interventions that prevent reported impairments, thus improving the quality of life of these patients.

## 5. Conclusions

Lung cancer survivors show an increase in symptomatology and physical deconditioning after surgery, which is maintained one month after surgery and could disturb the functionality and quality of life of these patients.

## Figures and Tables

**Table 1:** Characteristics of the sample before surgery.

Variables		Values (n = 88)
Sex (% male)		60.2
Age (years)		59.33 ± 13.63
BMI (kg/m^2^)		26.84 ± 4.42
Charlson index		4.55 ± 2.51
FEV1%		80.44 ± 23.63
Smoking habits n (%)	Non-smoker	24 (27.3)
	Smoker	6 (7.0)
	Ex-smoker	58 (65.7)
Smoking habits frequency n (%)	<5 cigarettes/day	36 (57.2)
	>5 cigarettes/day	28 (42.8)
Type of tumor n (%)	Squamous carcinoma	18 (20.5)
	Adenocarcinoma	41 (46.5)
	Metastatic	21 (23.0)
	Unclassified	8 (9.1)
Type of intervention n (%)	Segmental resection	36 (40.0)
	Lobectomy	50 (56.8)
	Pneumonectomy	2 (2.2)
Method of execution n (%)	VATS	58 (65.9)
	Thoracotomy	30 (34.1)
Type of anesthesia n (%)	General	88 (100)
	Local	0 (0)
Surgery duration (minutes)		206.18 ± 71.52
Post-operating complications n (%)		1 (1.1)
Length of stay (days)		6.82 ± 2.03

Data are expressed as mean ± SD or percentage (%). BMI: body mass index; FEV1%: forced expiratory volume in the first second; SD: Standard Deviation; VATS: Video-Assisted Thoracic Surgery.

**Table 2:** Analytic values pre- and post-surgery.

Variables	Pre-Surgery	Post-Surgery	*p*
Red blood cells	4.65 ± 0.54	4.14 ± 0.79	0.016 *
Hemoglobin	14.49 ± 4.08	11.82 ± 1.30	0.048 *
Hematocrit	41.62 ± 5.27	36.37 ± 3.81	0.013 *
Leukocytes	8.63 ± 4.69	12.49 ± 1.52	0.020 *
Platelet	242.66 ± 66.05	221.71 ± 49.58	0.217
pH	7.38 ± 0.03	7.37 ± 0.05	0.423
pCO_2_	43.46 ± 4.71	50.38 ± 16.80	0.031 *
pO_2_	105.31 ± 38.69	94.83 ± 32.41	0.247

Data are expressed as mean ± Standard Deviation; pCO_2_: carbon dioxide partial pressure; pO_2_: oxygen partial pressure. * *p* < 0.05.

**Table 3:** Pre- and post-surgery differences in symptoms and physical fitness in lung resection patients.

		Pre-Surgery (n = 88)	Post-Surgery (n = 88)	*p*
Symptoms				
Dyspnea		0.91 ± 2.09	1.61 ± 2.22	0.013 *
Fatigue		25.17 ± 19.05	30.65 ± 20.27	0.004 *
Cough		20.18 ± 2.24	18.38 ± 3.07	<0.001 **
Pain	Severity	2.89 ± 7.02	15.39 ± 9.91	<0.001 **
	Interference	4.08 ± 12.47	23.07 ± 20.61	<0.001 **
Physical Fitness				
Dynamometry	Dominant hand (N)	303.22 ±100.65	270.57 ± 105.82	<0.001 **
	Dominant leg (N)	114.41 ± 47.34	102.69 ± 49.26	0.001 *
UULEX	Time (seconds)	362.4 ± 282.74	108 ± 180.83	<0.001 **
	Dyspnea post-test	1 ± 2.04	1.23 ± 2.42	0.513
	Fatigue post-test	6.23 ± 2.48	5.62 ± 3.70	0.639
5STS	Time (seconds)	13.95 ± 9.63	17.87 ± 11.73	<0.001 **
	Dyspnea post-test	1.02 ± 2.23	2.22 ± 2.65	0.001 *
	Fatigue post-test	1.14 ± 2.20	1.37 ± 2.42	0.462

N: Newton; UULEX: unsupported upper limb exercise test; STS: Sit-to-Stand; * *p* < 0.05, ** *p* < 0.001.

**Table 4:** Pre-surgery and 1-month-after-surgery differences in symptoms and physical fitness in lung resection patients.

		Pre-Surgery (n = 88)	1-Month Follow-Up (n = 85)	*p*
Symptoms				
Dyspnea		1.1 ± 2.27	2.22 ± 2.94	0.007 *
Fatigue		25.66 ± 18.06	0.93 ± 0.76	<0.001 **
Cough		19.75 ± 2.68	19.42 ± 2.90	0.518
Pain	Severity	2.61 ± 6.61	6.63 ± 7.88	0.002 *
	Interference	3.32 ± 9.67	10.46 ± 16.80	0.005 *
Physical Fitness				
Dynamometry	Dominant hand (N)	309.23 ± 97.45	279.91 ± 94.98	0.001 *
	Dominant leg (N)	116.98 ± 48.51	128.95 ± 46.99	0.082
UULEX	Time (seconds)	404.35 ± 264.15	339.13 ± 229.97	0.023 *
	Dyspnea post-test	1.95 ± 2.85	3.05 ± 2.97	0.151
	Fatigue post-test	6.14 ± 2.47	5.81 ± 1.99	0.624
5STS	Time (seconds)	14.29 ± 11.03	14.45 ± 8.14	0.876
	Dyspnea post-test	1.48 ± 2.30	1.21 ± 0.41	0.529
	Fatigue post-test	1.18 ± 2.09	1.6 ± 2.63	0.242

N: Newton; UULEX: unsupported upper limb exercise test; STS: Sit-to-Stand; * *p* < 0.05, ** *p* < 0.001.

## Data Availability

The original contributions presented in this study are included in the article. Further inquiries can be directed to the corresponding author.
